# Identifying Barriers to Care in the Pediatric Acute Seizure Care Pathway

**DOI:** 10.5334/ijic.5598

**Published:** 2022-03-31

**Authors:** Michele C. Jackson, Alejandra Vasquez, Oluwafemi Ojo, Alexandra Fialkow, Sarah Hammond, Coral M. Stredny, Annalee Antonetty, Tobias Loddenkemper

**Affiliations:** 1 Division of Epilepsy and Clinical Neurophysiology, Department of Neurology, Boston Children’s Hospital, Harvard Medical School, Boston, MA, US; 2 Division of Child and Adolescent Neurology, Department of Neurology, Mayo Clinic, Rochester, MN, United States

**Keywords:** epilepsy, acute treatment and education, patient monitoring, care coordination, continuity of care, integrated care

## Abstract

**Objective:**

We aimed to describe the acute seizure care pathway for pediatric patients and identify barriers encountered by those involved in seizure care management. We also proposed interventions to bridge these care gaps within this pathway.

**Methods:**

We constructed a process map that illustrates the acute seizure care pathway for pediatric patients at Boston Children’s Hospital (BCH). The map was designed from knowledge gathered from unstructured interviews with experts at BCH, direct observation of patient care management at BCH through a quality improvement implemented seizure diary and from findings through three studies conducted at BCH, including a prospective observational study by the pediatric Status Epilepticus Research Group, a multi-site international consortium. We also reviewed the literature highlighting gaps and strategies in seizure care management.

**Results:**

Within the process map, we identified twenty-nine care gaps encountered by caregivers, care teams, residential and educational institutions, and proposed interventions to address these challenges. The process map outlines clinical care of a patient through the following settings: 1) pre-hospitalization setting, defined as residential and educational settings before hospital admission, 2) BCH emergency department and inpatient settings, 3) post-hospitalization setting, defined as residential and educational settings following hospital discharge or clinic visit and 4) follow-up BCH outpatient settings, including neurology, epilepsy, and primary care provider clinics. The acute seizure care pathway for a pediatric patient who presents with seizures exhibits at least twenty-nine challenges in acute seizure care management.

**Significance:**

Identification of care barriers in the acute seizure care pathway provides a necessary first step for implementing interventions and strategies in acute seizure care management that could potentially impact patient outcomes.

## Introduction

Epilepsy, one of the most common neurological conditions in childhood [1] has an estimated lifetime prevalence of 1% among children in the United States (US) [2]. As a chronic disorder, epilepsy poses a substantial burden on patients, caregivers, and the healthcare system. Children with seizures are more likely to experience comorbidities, such as intellectual and learning disabilities [2,3,4,5], language development delay [4], depression, anxiety, and autism [2,4]. For pediatric patients with epilepsy (PPWE) in the US, there is also an additional cost of $9,103.25 per year per person compared to those without epilepsy [6].

The Institute of Medicine addressed strategies to ameliorate the epilepsy burden and improve care quality with focused research initiatives [7]. Acknowledgement of these improvement recommendations reflects the importance of establishing an integrated epilepsy care approach to ensure excellent care. Nevertheless, current research provides evidence that gaps continue to exist at many levels in seizure management [8,9,10], prompting the need for action. Our primary aim was to describe the acute seizure care pathway as a process map diagram for pediatric patients and identify the gaps in care encountered by those involved in seizure care management. Our secondary aim was to propose interventions to bridge these care gaps within the acute seizure care pathway. We also conducted a review of current studies to further support the existence of the identified care barriers as outlined in the process map and provide examples of interventions from the literature that have already been explored or implemented.

## Methods

We constructed a process map that illustrates the acute seizure care pathway for pediatric patients at Boston Children’s Hospital (BCH), a tertiary care center in Boston, Massachusetts. Within the map, we identified care gaps encountered by four key stakeholders: 1.) patients with seizures, 2.) caregivers (parents, guardians, patient family members, friends), 3.) residential and educational organizations (i.e. school, daycare) and their professionals (i.e. teachers, teacher aides, nurses, chaperones) and 4.) clinical care teams at all training levels (i.e. primary care providers, neurologists, epileptologists, resident and attending physicians). We proposed interventions to address these challenges and developed corresponding solutions to the gaps identified by the map (***Tables 1*** and ***2***).

**Table 1:** **Acute Seizure Care Pathway Care Gaps and Interventions.** Summary of twenty-nine care gaps along the acute seizure care pathway, evidence-based interventions to bridge these gaps, and the care setting location for the implementation of the interventions.


GAP		INTERVENTION	IMPLEMENTATION LOCATION

1	Seizure onset not recognized by another individual	Implement patient seizure monitoring system to [43,48,52,53] Equip patients with a customized multimodal seizure detection device Alert caregivers of seizure Transmit physiological data from device to EMR Provide clinicians with objective quantifiable clinical data	Emergency Department Inpatient Outpatient Post-Hospitalization

2	SAP not available	Physician prescribes SAP and RM Hospital and clinic staff train caregivers on SAP and RM administration through “hands-on” seizure simulation modules and mannequins [24,26] Provide caregivers with physical reminders of SAP and RM instructions, such as refrigerator magnets and cards [28,36] Implement RM administration methods that are preferred by users [72,73] Implement urgent epilepsy care clinic access to: Provide caregivers with direct access to additional medical resources, such as a nurse navigator or care coordinator [60,61] Provide caregivers with direct access to psychosocial counseling [27,28] Implement electronic care coordination system to: Provide caregivers with direct access to additional medical resources, such as a nurse navigator or care coordinator [60,61] Facilitate dissemination of SAP and RM Schedule SAP and RM training Track SAP and RM training and sharing of SAP and RM among caregivers and outside institutions	

3	SAP not implemented		

4	RM not available		

5	RM not administered		

6	Drug not administered through proper route and dosage		

7	EMS does not administer RM	Standardize EMS seizure protocols with weight-based dosing [30,35] Train EMS on seizure detection and diagnosis of prolonged seizure Train EMS on RM administration through “hands-on” seizure simulation modules and mannequins [31] Implement RM administration methods that are preferred by users [72,73] Equip EMS units with RM and second-line therapy Refresher EMS courses on pediatric care and management [31]	Pre-Hospitalization

8	EMS does not administer the correct dosage of rescue medication		

9	Staff delay	Implement seizure action code to alert [37,38]: SE and seizure intervention teams Pharmacy SE and seizure teams Implement pharmacy systems to ensure medication availability and centralization of RM on each hospital floor [37]	Emergency Department Inpatient

10	Pharmacy delay		

11	ASM delay		

12	ASM unavailable		

13	Deviation from the treatment protocol	Standardize SE and seizure algorithms with weight-based doses [30,35] Standardize SE and seizure algorithms in pre and in-hospital care settings to assure algorithm adherence and continuation of care [33,34,35,36,37] Integrate SE algorithm and SAP in the electronic physician order set [36,37,38] Standardization of clinic notes, detailing seizure history and events [37] Train ED and inpatient staff on SE and seizure algorithms through “hands-on” seizure simulation modules and mannequins [34] Require all clinicians to watch an audiovisual seizure treatment training module before inpatient service [37] Provide clinicians with physical reminders of SE and seizure algorithms, such as cards [36]	

14	Route of administration difficulties		

15	Delay in obtaining EEG results if the diagnosis is unclear	Implement advanced EEG seizure detection technology to prevent EEG delay across EMS and inpatient settings Improve the clinical process to decrease the time from seizure onset to placement of EEG technology [39]	

16	Patient does not bring a personal seizure diary and medication log	Implement patient seizure monitoring system to [43,48,52,53]: Equip patients with customized multimodal seizure detection devices Alert caregivers Transmit physiological data to EMR Provide clinicians with objective quantifiable clinical data Implement EMR-integrated personal seizure diary and medication log to: Transmit seizure and medication data directly to EMR-integrated visualization system Provide clinicians with objective quantifiable clinical data	Inpatient Outpatient Post-Hospitalization

17	Patient not given seizure diary and medication log		

18	SAP not prescribed or SAP updated	Physicians prescribe SAP and RM Hospital and clinic staff train caregivers on SAP and RM administration through “hands-on” seizure simulation modules and mannequins [24,26] Provide caregivers with physical reminders of SAP and RM instructions, such as refrigerator magnets and cards [28,36] Implement RM administration methods that are preferred by users [72,73] Implement urgent epilepsy care clinic to: Provide caregivers with direct access to additional medical resources, such as a nurse navigator or care coordinator [60,61] Provide caregivers with direct access to psychosocial counseling [27,28] Implement electronic care coordination system to: Provide caregivers with direct access to additional medical resources, such as a nurse navigator or care coordinator [60,61] Facilitate dissemination of SAP and RM Schedule SAP and RM trainings and re-fresher trainings Track SAP and RM training and sharing of SAP and RM among caregivers and outside institutions	

19	Caregiver not trained on SAP		

20	RM not prescribed for patient		

21	Caregiver not trained on RM administration		

22	Caregiver does not schedule appointment	Implement electronic care coordination system to: Provide caregivers with direct access to additional medical resources, such as a nurse navigator or care coordinator [60,61] Schedule and reschedule appointments Send appointment reminders	

23	Patient does not attend appointment		

24	Caregiver does not fill RM prescription	Implement electronic care coordination system to: Provide caregivers with direct access to additional medical resources, such as a nurse navigator or care coordinator [60,61] Track RM prescriptions and refills Facilitate dissemination of RM and SAP Track sharing of RM and SAP among patient caregivers and outside institutions Equip outside institutions with trained medical staff that can administer RM and SAP [24,26]	

25	Caregiver does not give RM to outside institutions		

26	Outside institution cannot legally administer RM		

27	Outside institution not trained on RM administration		

28	Caregiver does not provide SAP to outside institutions		

29	Outside institution not trained on SAP		


**EMR:** Electronic Medical Record, **PCP:** Primary Care Provider, **SAP:** Seizure Action Plan, **RM:** Rescue Medication, **SE:** Status Epilepticus, **EMS:** Emergency Medical Services, **ASM:** Anti-Seizure Medication, **ED:** Emergency Department.

**Table 2:** **Acute Seizure Care Pathway Interventions and Implementation Care Group.** Summary of twenty-five proposed interventions delineated by the key clinical and patient family care stakeholders.


INTERVENTION		IMPLEMENTATION CARE GROUP

A	Implement patient seizure monitoring system to [43,48,52,53]: Equip patients with a customized multimodal seizure detection device Alert caregivers of seizure onset Transmit physiological data from device to EMR Provide clinicians with objective, quantifiable clinical data	Hospital, Emergency Physician, Neurologist, Epileptologist, Patient Family, Insurance

B	Physician prescribes SAP and RM	Emergency Physician, Neurologist, Epileptologist, Clinic Staff

C	Implement RM administration methods that are preferred by users [72,73]	

D	Hospital and clinic staff train caregivers on SAP and RM administration through “hands-on” seizure simulation modules and mannequins [24,26]	

E	Provide caregivers with physical reminders of SAP and RM instructions, such as refrigerator magnets and cards [28,36]	

F	Implement inpatient seizure action code to alert [37,38]: SE and seizure intervention teams Pharmacy SE and seizure teams	

G	Standardize SE and seizure algorithms with weight-based doses [30,35]	

H	Standardize SE and seizure algorithms in pre- and in-hospital care settings to assure algorithm adherence and continuation of care [33,34,35,36,37]	

I	Integrate SE algorithm and SAP in the electronic physician order set [36,37,38]	

J	Standardization of clinic notes, detailing seizure history and events [37]	

K	Train ED and inpatient staff on SE and seizure algorithms through “hands-on” seizure simulation modules and mannequins [34]	

L	Require all clinicians to watch an audiovisual seizure treatment training module before inpatient service [37]	

M	Provide clinicians with physical reminders of SE and seizure algorithms, such as cards [36]	

N	Improve the clinical process to decrease the time from seizure onset to placement of EEG technology [39]	

O	Implement advanced EEG seizure detection technology to prevent EEG delay across EMS and inpatient settings	Hospital, Emergency Physician, Neurologist, Epileptologist, Clinic Staff

P	Implement pharmacy systems to ensure medication availability and centralization of RM on each hospital floor [37]	

Q	Implement EMR-integrated personal seizure diary and medication log to: Transmit seizure and medication data directly to EMR-integrated visualization system Provide clinicians with objective quantifiable clinical data	

R	Implement urgent epilepsy care clinic access to: Provide caregivers with direct access to additional medical resources, such as a nurse navigator or care coordinator [60,61] Provide caregivers with direct access to psychosocial counseling [27,28]	

S	Implement electronic care coordination system to: Provide caregivers with direct access to additional medical resources, such as a nurse navigator or care coordinator [60,61] Facilitate dissemination of SAP and RM Schedule SAP and RM training Track SAP and RM training and sharing of SAP and RM among caregivers and outside institutions Schedule and reschedule appointments Send appointment reminders	

T	Equip EMS units with RM and second-line therapy	EMS, Emergency Physician, Neurologist, Epileptologist, Clinic Staff

U	Standardize EMS seizure protocols with weight-based dosing [30,35]	

V	Train EMS on seizure detection and diagnosis of prolonged seizure	

W	Train EMS on RM administration through “hands-on” seizure simulation modules and mannequins [31]	

X	Refresher EMS courses on pediatric care and management [31]	

Y	Equip outside institutions with trained medical staff that can administer RM and SAP [24]	Outside Institutions


**EMR:** Electronic Medical Record, **SAP:** Seizure Action Plan, **RM:** Rescue Medication, **SE:** Status Epilepticus, **EMS:** Emergency Medical Services, **ASM:** Anti-Seizure Medication, **ED:** Emergency Department.

**Figure 1:**
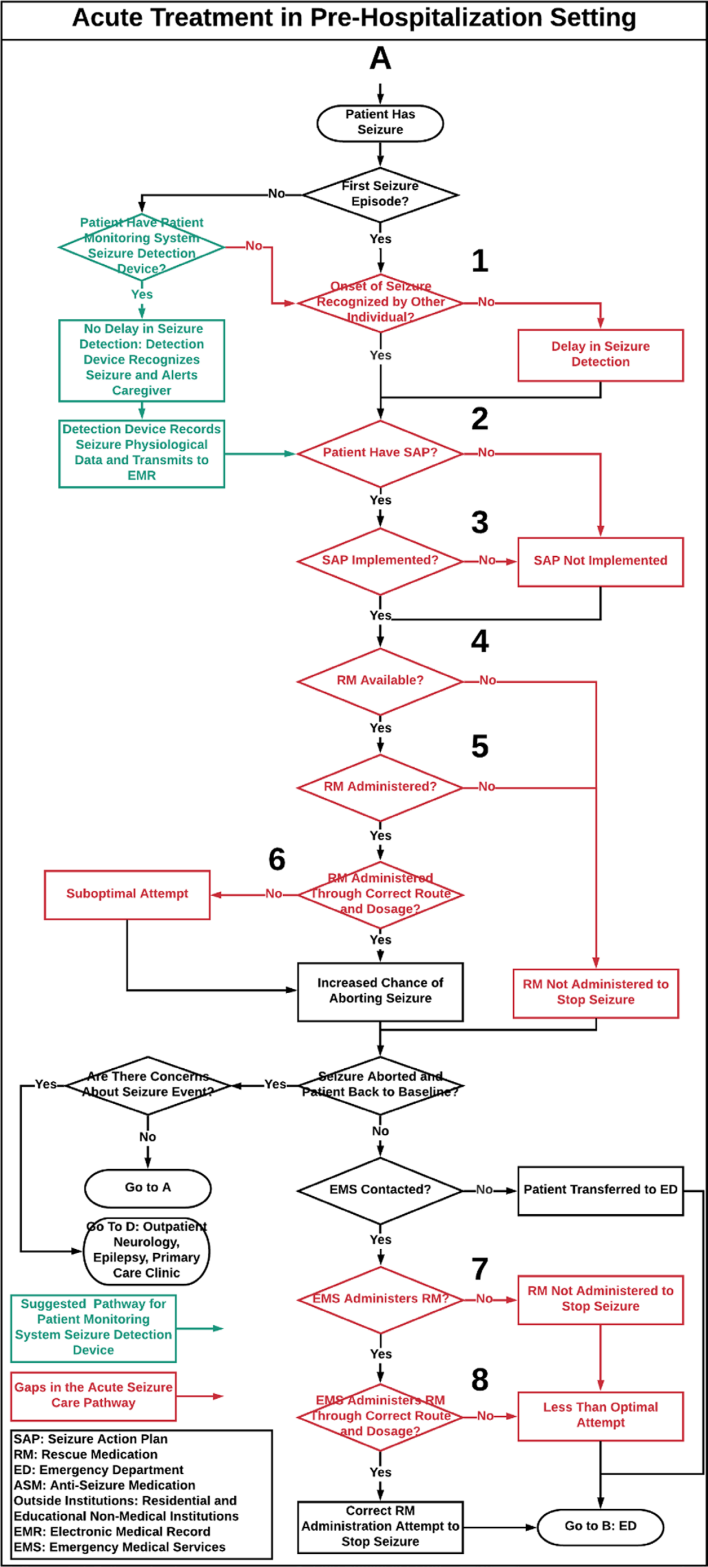
**Acute Treatment in Pre-Hospitalization Setting.** Acute seizure care process map that illustrates the flow of epilepsy care management in a tertiary hospital through all care steps that a patient with a seizure may encounter from pre-hospitalization to the ED. Numbers on the process map identify care gaps in acute seizure care management and refer to the corresponding Table 1, which proposes strategies to bridge these gaps. **SAP:** Seizure Action Plan, **RM:** Rescue Medication, **ED:** Emergency Department. **Red:** Gaps in Seizure Care Management, **Green:** Does not currently exist as a process.

**Figure 2:**
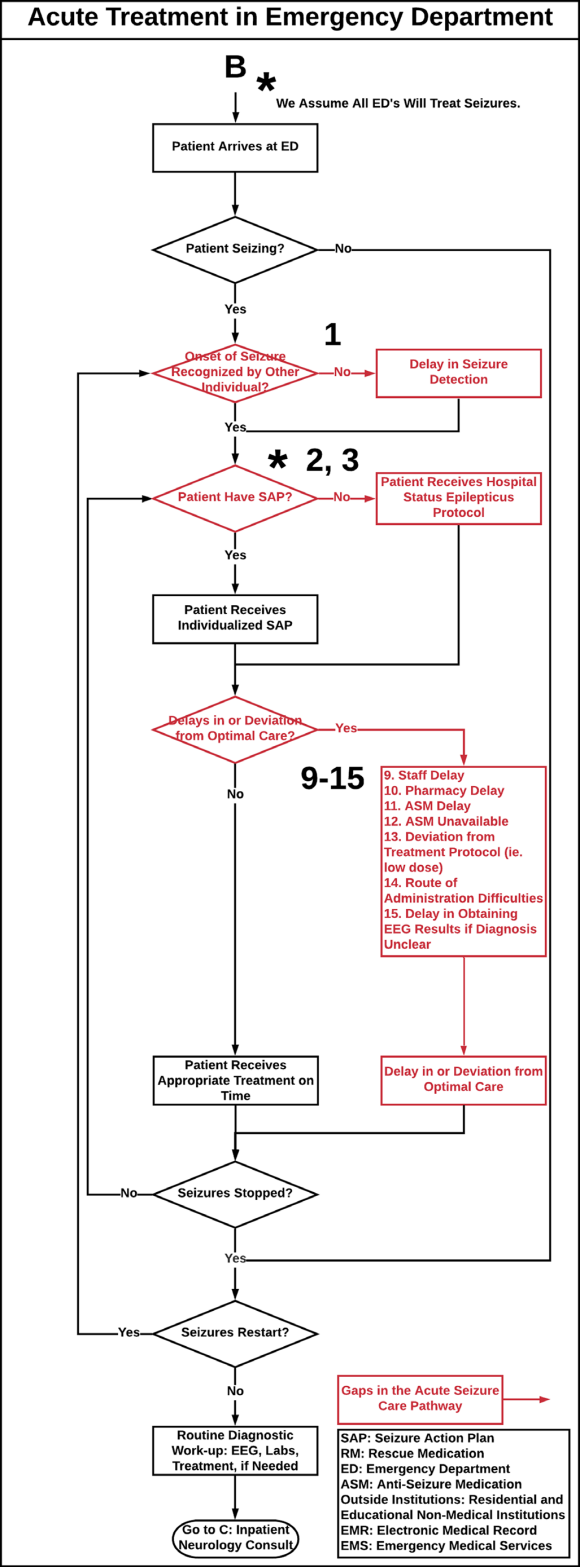
**Acute Treatment in Emergency Department.** Acute seizure care process map that illustrates the flow of epilepsy care management in a tertiary hospital through all care steps that a patient with a seizure may encounter from the ED to inpatient care settings. Numbers on the process map identify care gaps in acute seizure care management and refer to the corresponding Table 1, which proposes strategies to bridge these gaps. **SAP:** Seizure Action Plan, **RM:** Rescue Medication, **ED:** Emergency Department. **Red:** Gaps in Seizure Care Management, **Green:** Does not currently exist as a process.

**Figure 3:**
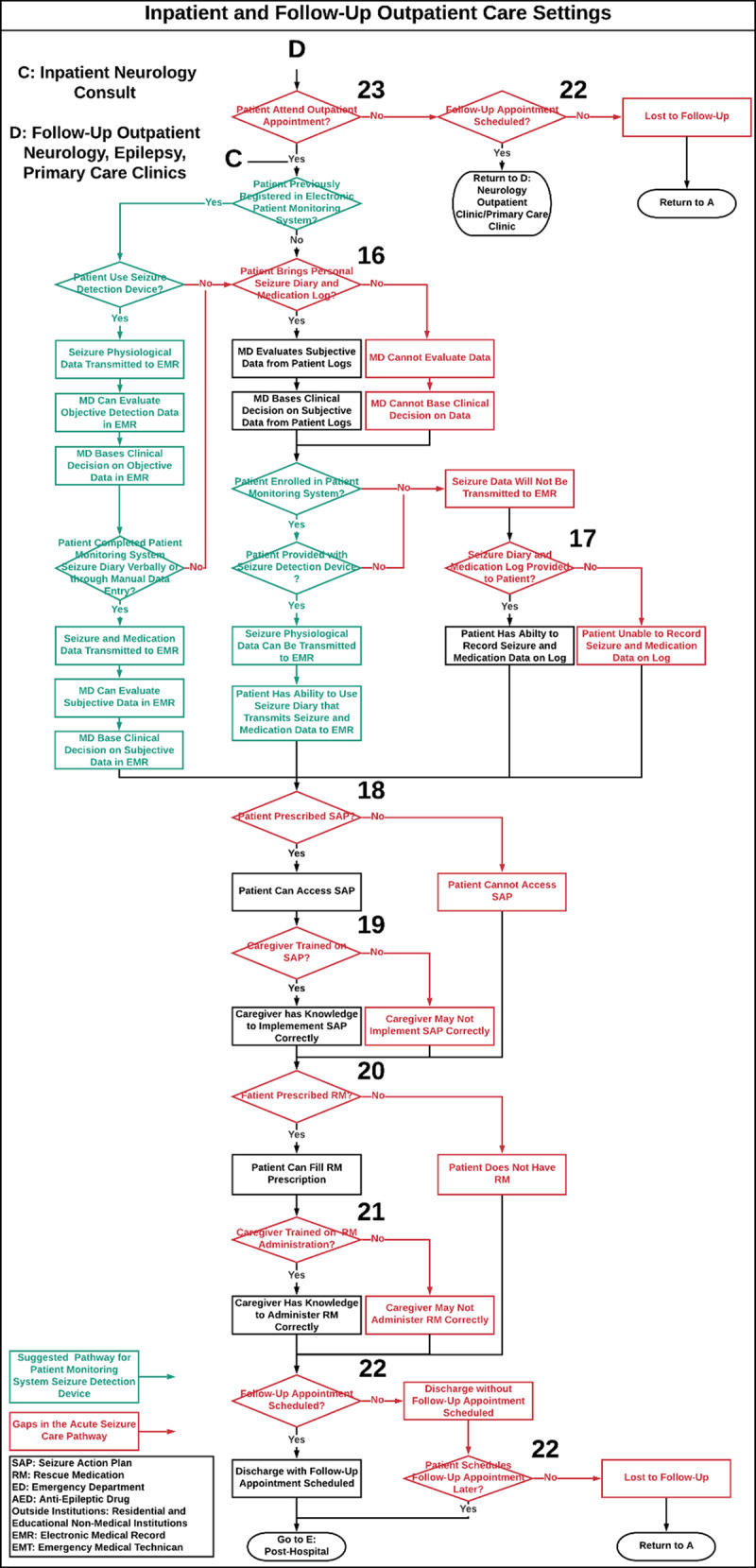
**Inpatient and Follow-Up Outpatient Care Settings.** Acute seizure care process map that illustrates the flow of epilepsy care management in a tertiary hospital through all care steps that a patient with a seizure may encounter from the ED to the inpatient and follow-up outpatient neurology, epilepsy, and primary care clinic settings. Numbers on the process map identify care gaps in acute seizure care management and refer to the corresponding Table 1, which proposes strategies to close these gaps. **SAP:** Seizure Action Plan, **RM:** Rescue Medication, **ED:** Emergency Department, **ASM:** Anti-Seizure Medication, **Outside Institutions:** Residential and Educational Non-Medical Institutions, **EMR:** Electronic Medical Record, **EMS:** Emergency Medical Services. **Red:** Gaps in Seizure Care Management, **Green:** Does not currently exist as a process.

**Figure 4:**
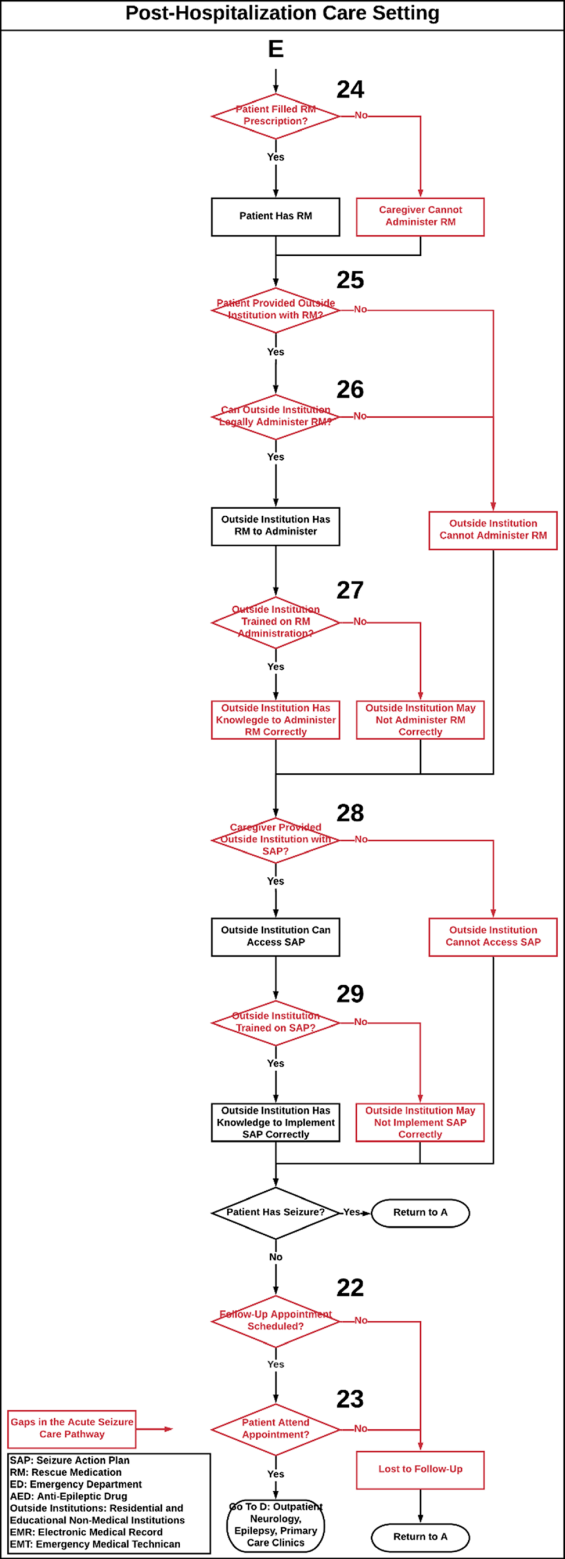
**Post-Hospitalization Care Setting.** Acute seizure care process map that illustrates the flow of epilepsy care management in a tertiary hospital through all care steps that a patient with a seizure may encounter from the follow-up outpatient neurology, epilepsy, and primary care clinic settings to post-hospitalization care settings. Numbers on the process map identify care gaps in acute seizure care management and refer to the corresponding Table 1, which proposes strategies to close these gaps. **SAP:** Seizure Action Plan, **RM:** Rescue Medication, **ED:** Emergency Department, **ASM:** Anti-Seizure Medication, **Outside Institutions:** Residential and Educational Non-Medical Institutions, **EMR:** Electronic Medical Record, **EMS:** Emergency Medical Services. **Red:** Gaps in Seizure Care Management, **Green:** Does not currently exist as a process.

The seizure care process map outlines the care of a patient with seizures through the following settings: 1.) pre-hospitalization, defined as emergency medical services (EMS) that provide urgent and immediate care in the out-of-hospital settings before BCH admission (***Figure 1***), 2.) BCH ED (***Figure 2***) and inpatient settings (i.e. neurology, epilepsy and intensive care units, ***Figure 3***), 3.) post-hospitalization, defined as residential and educational settings (i.e. home, school, daycare) following hospital discharge or outpatient clinic visit (***Figure 4***) and 4.) follow-up BCH outpatient settings, including neurology, epilepsy, and primary provider clinics (***Figure 3***).

The pathway was designed using data gathered through five avenues: 1) four unstructured interviews with neurology and epilepsy ambulatory and inpatient healthcare providers as part of a neurology quality improvement (QI) project with the aim of designing a seizure frequency, clinical history and medication electronic medical record (EMR) integrated-visual interface for patients with seizures. Providers were asked to discuss challenges they face in both inpatient and outpatient settings in treating patients with seizures regarding training for those involved in seizure care management, clinical data collection and follow-up visit management. Their insight was used to identify gaps in care in the acute seizure care pathway and provide strategies that may help to bridge the care gaps. The interviews provided understanding to the care challenges that clinicians and caregivers experience in all seizure care settings.; 2) experience and direct observation of epilepsy patient care management from a QI initiative that implemented an EMR-integrated electronic seizure diary at BCH and enrolled 157 PPWE into the diary in the BCH neurology and epilepsy outpatient and inpatient settings [11]. The EMR QI project provided information regarding care barriers faced by caregivers and clinicians in the follow-up outpatient neurology, epilepsy, and primary care clinics.; 3) findings from a QI study at BCH that implemented a standardized discharge template for PPWE through three Plan-Do-Study-Act (PDSA) cycles and examined the readmissions of these patients through the emergency department [12,13,14]. The readmissions QI project provided information regarding care barriers faced by caregivers and clinicians in the follow-up outpatient neurology, epilepsy, and primary care clinics.; 4) findings from a prospective cross-sectional observational study at BCH that analyzed pre-hospital seizure rescue medication (RM) use, caregiver knowledge and comfort, and prescription patterns in PPWE. The observational study on RM use provided insight into gaps in care in the pre- and post-hospitalization settings.; 5) experience and findings on status epilepticus (SE) outcomes and treatment strategies from the pediatric Status Epilepticus Research Group (pSERG), a multi-site international consortium that conducts an ongoing prospective observational study at twenty-five centers (*www.pserg.org*) [10]. The pSERG observational study provided insight into pre-hospitalization, ED and inpatient neurology care settings.

We conducted a literature review to provide further evidence for seizure care barriers and to present examples of strategies currently implemented in epilepsy management. From our literature review and development of the map, we proposed interventions and strategies to bridge these gaps at BCH.

Approval from the BCH Institutional Review Board (IRB) was obtained before data acquisition for the visual interface and seizure diary QI initiatives and research studies. IRB approval was not required for map creation and literature review.

The map was built using Lucidchart (Lucid Software Inc., Salt Lake City, Utah.) and reviewed for accuracy at BCH by two neurologists, one epileptologist, and three QI consultants and analysts.

## Results

### Acute Seizure Care Pathway

The acute seizure care pathway for a pediatric patient who presents with a seizure is shown in ***Figures 1***, ***2***, ***3***, and ***4***. Results are presented according to the following settings: 1) pre-hospitalization (***Figure 1***), 2) ED (***Figure 2***) and inpatient (***Figure 3***), 3) post-hospitalization (***Figure 4***) and 4) follow-up outpatient clinics (***Figure 3***). We identified twenty-nine potential gaps in the acute seizure care pathway experienced in seizure management by patients with seizures, caregivers, residential and educational organizations, and their professionals and care teams (***Tables 1*** and ***2***).

### Pre-Hospitalization Setting

The pathway starts with a seizure occurrence in the pre-hospitalization setting and details the steps of acute seizure care from seizure onset to the transfer of the patient to either of three settings: the ED, inpatient, or outpatient setting. The pathway first differentiates whether the seizure was recurrent and an individual recognized seizure onset. The map addresses RM availability and utilization, seizure action plan (SAP) implementation, and EMS utilization (***Figure 1***). RM is diazepam, lorazepam, and midazolam used for emergency seizure rescue. Patients who do not travel to the ED may instead be seen in the acute neurology, epilepsy, or primary care outpatient clinics (***Figure 3***).

### ED and Inpatient Neurology Care Settings

Following seizure onset in the pre-hospital setting, the patient may proceed to the ED and inpatient settings. The map integrates inpatient seizure resolution or recurrence and outlines subsequent inpatient care steps. In the ED and inpatient setting, the map addresses inpatient seizure onset detection, SAP utilization, and potential inpatient acute seizure care delays (***Figures 2*** and ***3***). Under inpatient neurology care, the pathway incorporates RM prescriptions, SAP and RM education, seizure and medication diary utilization, and follow-up appointment scheduling. Following discharge from the inpatient setting or ED, the patient proceeds to the post-hospitalization setting (***Figure 4***).

### Post-Hospitalization Care Setting

After the patient is discharged from the hospital, the map outlines steps that a caregiver and patient undergo regarding filling a RM prescription, and SAP and RM dissemination among residential and educational settings. The pathway addresses whether these institutions can administer RM and if they receive training (***Figure 4***).

### Follow-Up Outpatient Neurology, Epilepsy, and Primary Care Clinics

In the follow-up outpatient setting, the patient may proceed to the neurology, epilepsy, or primary outpatient clinics. The map recognizes patients lost to follow-up, scheduled outpatient appointment attendance, and seizure and medication diary adherence. The map addresses physician assessment of seizure and medication history, SAP and RM education, and the implementation of medication and seizure diaries (***Figure 3***).

### Four Domains: Acute Treatment, Patient Monitoring, Care Integration and Prevention of Hospital Utilization

This acute seizure care process map identifies twenty-nine gaps within the pathway (***Figures 1***, ***2***, ***3*** and ***4***, ***Tables 1*** and ***2***). Potential interventions to overcome these challenges fall under four domains: 1) acute treatment, 2) patient monitoring, 3) care integration and follow-up, and 4) prevention of hospital utilization. These strategies aim to close the loop between caregivers and care teams through the integration of multidisciplinary communication approaches, patient monitoring, and coordination systems, and enhanced training modules for seizure management (***Tables 1*** and ***2***).

### Acute Treatment

Prompt seizure termination is the central pillar in epilepsy treatment strategies and thus an important opportunity for intervention in acute seizure management. Rapid administration of benzodiazepines (BZDs) as first-line treatment is supported by class I evidence [15] and is an essential step independent of the location of seizure onset. Considering that the majority of seizures start out-of-hospital [9], timely utilization of BZD RM in the pre-hospital setting may prevent prolonged seizure duration [16], a determinant of mortality [17,18]. Pre-hospital BZD RM utilization is associated with a shorter duration of generalized convulsive SE [19], a lower probability of recurrent seizures in the ED [19] and decreased ED visits [20], while the absence of pre-hospital RM is correlated with a higher probability of ED visits and unplanned hospitalizations. Despite evidence of adverse outcomes, delay in pre-hospital RM persists in children both with and without a history of seizures [21]. Although a history of seizures or SE often precludes a lack of RM, studies on convulsive SE treatment demonstrated that pre-hospital RM was administered in 34% [22] and 44.4% [9] of children with a seizure history and in 33.3% [9] with prior SE. Hence, a focus on timely and appropriately dosed medication remains a critical first step towards care improvements.

### Acute Treatment: Residential and Educational Settings

Pre-hospital RM availability and correct usage at seizure onset are two of the most opportune gaps for intervention in acute seizure management, as identified by the map (***Table 1***, Gap: 4–8, ***Table 2***: B & Y). Caregivers, paramedics, and clinicians may experience a variety of barriers to ensure RM accessibility and proper utilization. Such barriers include obtaining medication prescriptions, high costs, and coordinating medication dissemination and training for caregivers and staff at out-of-hospital settings including home, school, daycare, camp, and mobile settings (i.e. car, plane, train). For instance, in PPWE, 87% of patients were prescribed a RM [23]. Of these patients, 2.3% did not have medication available at home, while 16.1% did not have medication available outside the home [23]. Furthermore, 7.5% of caregivers reported being the sole individual with RM administration knowledge [23]. RM cost was reported by 4.6% of caregivers to be a deterrent to attaining medication [23], while others were not able to administer medication simply because the prescription was not refilled. To assist caregivers in managing RM accessibility, an electronic care coordination system could be integrated among healthcare institutions. A coordinator or nurse navigator could assist with post-seizure follow-up to ensure RM availability, address caregiver questions, and provide counseling to troubleshoot seizure care difficulties.

RM availability and utilization in educational settings are limited due to school legal restrictions, preferences, inadequately trained staff, and caregiver concerns. Although seizures constitute 16% of school-based EMS calls and are the third most frequently reported school emergency, an analysis of nurse confidence levels revealed that 33% of school nurses do not feel comfortable treating seizures. Nineteen percent of schools that were asked to utilize RM refused due to legal restrictions, privacy concerns, and doubts that staff could learn how to administer medication. Parents recounted that the schools’ RM administration concerns had adverse effects on their family, such that caregivers were not able to attend their work or school, kept their child at home, or attended school with their child. Seventeen percent of parents did not ask their school to administer rectal diazepam due to not wanting the school to administer medication, fear of harm, unknowing that they could ask the school to administer medication, and beliefs that staff could not be trained. RM administration mannequin simulations increased pre-school teacher self-confidence and willingness in administrating rectal RM and significantly reduced RM administration errors [24]. Simulation-based training modules and the integration of SAPs in emergency protocols may address these concerns, instill confidence and reduce errors.

As indicated in the map, RM may not be administered appropriately by caregivers, despite access. Of patients that were prescribed a RM, only 60.9% of the caregivers received RM training [23]. A seizure home management study discovered that caregivers did not utilize rectal RM due to concerns regarding seizure recognition and not knowing how to administer RM [25]. In caregivers that claimed to have never experienced problems administering RM, investigators observed high-risk handling errors when caregivers administered medication to mannequins [26]. Caregivers have demonstrated higher errors in rectal than in buccal application, and previous RM use was not a predictor for the number of errors [26], supporting the need for seizure simulated mannequin training modules (***Table 2***: D).

Comprehensive integration of interactive seizure simulation RM training curriculums, and SAPs in clinical care could mitigate seizure detection and RM errors among caregivers in out-of-hospital settings (***Table 2***: B-D). The guidance and experience gained from these programs could foster caregiver self-confidence in seizure management. SAPs have been successful in educating families on seizure and RM protocols [23]. Families who recalled receiving a SAP were significantly more knowledgeable regarding ASM name and administration timing and were more likely to have RM available at school [23]. QI interventions decreased yearly ED and inpatient hospitalizations for PPWE by implementing focused strategies, such as magnets with RM use instructions, a simplified SAP, and access to urgent neurology care [27,28]. Caregivers have requested more hands-on and refresher trainings, SAPs, and educational materials to provide to other caregivers at residential and educational institutions [23].

### Acute Treatment: Emergency Medical Services

As there are significant systematic delays in the pre-hospital setting [16,29], revising EMS seizure protocols and ensuring that paramedics are empowered to deliver timely [30] and appropriate RM dosing and second-line therapy may optimize acute seizure management (***Table 1***, Gap: 7, 8, ***Table 2***: T-X). EMS management may be improved by standardizing and expanding protocols to administration of midazolam [30] and second-line therapy with appropriate weight-based doses and improving paramedic-specialized training [30]. Studies have established that there may also be room for improvement in emergency medical technician (EMT) training curricula [31,32].

### Acute Treatment: Inpatient Setting

Efforts to standardize and integrate protocols and medication order sets with the electronic medical record (EMR) may improve implementation of treatment SE algorithms and optimize standard of care (***Table 1***, Gap: 9–15). In the in-hospital setting, time from convulsive SE onset to ASM administration and escalation from one ASM class to another is delayed, and delay correlated with prolonged SE duration [9]. Time to inpatient treatment delays and variability is reduced through an EMR-integrated standardized SE protocol [33,34,35,36,37] with weight base doses [30,35], automated order sets [36,37,38], a seizure alarm code [37,38] and centralization of RM and supplies on each hospital floor [37] (***Table 2***: E, G, H, I). Through QI interventions and standardization of intranasal (IN) midazolam as first-line treatment, patients treated with a benzodiazepine within 10 minutes of seizure onset increased from 39% to 79% [37]. The implementation of a linear midazolam-based protocol also yielded 93% adherence to first-line therapy [21,35]. Through a standardized in-hospital seizure protocol and EMR-integrated automated order set, time to treatment was decreased by greater than 50% for first-line drugs [36]. Additional training proved effective in ensuring proficiency on a standardized SE algorithm.

In conjunction with QI interventions, audiovisual SE module training before inpatient service reduced first-line treatment delay significantly [37]. Further improvements may be achieved by implementation of an inpatient ‘seizure code’ mechanism to alert seizure and SE intervention teams and thus may reduce time to treatment (***Table 2***: F) [37,38].

Additionally, availability of advanced electroencephalogram (EEG) technology and highly-trained ED physicians and epileptologists may ameliorate diagnostic and treatment delays [39]. Outfitting remote EEG technology with EMS agencies could advance SE detection and decrease pre-hospital treatment delay (***Table 2***: N & O).

Even with the establishment of standardized SE algorithms, RM dose and drug choice in clinical practice differ from SE therapy guideline recommendations (***Table 2***: G & H). Under-dosing of BZDs in both pre- and in-hospital settings remains a problem in seizure management [16,40,41] and is associated with a higher probability of ED visits and unplanned hospitalization. Administration of multiple doses of BZDs, despite BZDs failing to control SE, lead to delayed escalation from first to second and third-line therapies [9,22,41,42]. Standardizing a transition protocol for patients from pre-hospital to ED and inpatient setting would improve adherence to treatment algorithms among settings and prevent multiple administrations of BZDs. Implementation of effective and fail-safe RM delivery methods in seizure treatment algorithms has the potential to increase RM utilization and protocol adherence.

### Patient Monitoring

Warning systems may provide several pieces of information that may help to care for patients with refractory seizures and SE: 1) a seizure diary, 2) sensor devices that automatically receive physiological input from patients, 3) seizure alarm systems that may be able to alert providers and caregivers of a seizure, 4) data visualization systems that graphically display seizure event and medication data in the EMR [43].

In a non-hospital setting, detection of and response to a seizure proves challenging (***Table 1***, Gap: 1–6, ***Table 2***: A). As wearable devices and machine learning become ubiquitous, they represent an increasingly robust option to revolutionize the management of health conditions, the diagnosis of medical problems, and the interactions that occur between doctors and their patients. The incorporation of seizure detection devices and a more comprehensive, closed-loop monitoring system into standard care may improve outcomes at various stages of the pathway.

Initially, seizure onset, especially atypical and non-motor onset seizure presentations, can be difficult to recognize. Prompt seizure recognition is incredibly important in the initiation of treatment. When an epilepsy monitoring unit incorporated one-to-one bedside observers during intracranial stereotactic EEG monitoring, sitters successfully lowered the rate of unrecognized seizures from 33.3% to 15%. Without sitters, motor seizures occurred without recognition, while with sitters, only seizures with a focal onset and impaired awareness went unnoticed [44]. Without intervention, the likelihood of adverse outcomes, including sudden unexpected death in epilepsy (SUDEP), increases [45]. Though the dedication of an observer to patients seems unrealistic beyond the walls of the monitoring unit, careful attention to and recognition of seizures spurs treatment and may improve outcomes.

Devices, however, may become reliable witnesses to help caregivers detect and respond to seizures. Particular individuals, such as teachers and other witnesses, may be unable to recognize seizure onset [46] in the absence of training and guidance from healthcare professionals or parents, and in the absence of technological alert devices (***Table 1***, Gap: 1, ***Table 2***: A). Many families change their sleep behaviors to co-sleep or watch their child sleep to respond to nocturnal seizures [47], but this approach becomes increasingly unrealistic as the child ages and as the sleep hygiene of family members deteriorates. In a group of patients with newly diagnosed epilepsy, patient families for whom a monitoring device was utilized saw a reduction in fear of further seizures and co-sleeping when compared to a group without monitoring [48]. The addition of monitoring devices to a patient’s care may increase the likelihood of event recognition, particularly in settings where ordinary seizure detection may be compromised. Such wearable devices can contain multiple sensors [43,49,50,51]. Sensor combinations in multi-modal monitoring devices may also improve detection of seizures, which can, in turn, improve response and outcomes.

Beyond seizure recognition, devices may decrease caregiver response time to seizures. Devices may alarm or send alerts to effect more rapid care [52], specifically to prompt RM use or to call for emergency medical attention. This warning and reminder system may be useful in a pre-hospital setting, especially when a caregiver’s fears and anxieties related to a seizure can hinder a quick response. Alerts act as corrective feedback to a stimulus in a closed-loop warning system, which continuously collects, transmits, and processes data. When a seizure occurs, an alert serves as a response to provoke specific further action, such as the administration of an RM or to call for personnel, to cause a return to baseline [53]. Such a device-based closed-loop warning system represents a potential to ensure continuity of care for PPWE [43].

With regards to long-term seizure management, devices can provide critical data on seizure frequency, timing, and semiology based on physiological signals (***Table 1***, Gap: 16, 17). Such data can be reviewed by the epilepsy team to gain a deeper understanding of a patient’s seizures, identify trends that may aid in seizure prediction, and develop a more comprehensive seizure action plan to treat events. Such an analytical monitoring process occurs in EEG units in many hospitals, which results in concrete treatment changes in approximately 79% of admissions [54]. EEG monitoring with routine 10–20 EEG lead setup, however, is unrealistic for everyday seizure detection, due to feasibility and potential stigma. With a less conspicuous device-based closed-loop system, however, seizure activity can be more easily and less expensively monitored. This enables patients’ families to become more prepared for seizure onset and gain confidence in their ability to respond to seizures appropriately. VNS therapy resulted in a higher quality of life among patients with pharmaco-resistant epilepsy when compared to patients receiving best medical practice alone [55]. This represents the potential for devices to better support patients.

Devices can be integrated into a larger data visualization system that can combine physiological, patient, and caregiver-reported seizure and medication data, and a SAP in a centralized EMR [43]. This platform can be viewable and accessible by patients and their care team to increase communication between visits. A system that combines multiple data streams from clinical and device-related healthcare variables has the potential to streamline individualized care and improve seizure outcomes and quality of life (***Table 2***: Q).

## Discussion

### Care Integration and Outpatient Follow-Up

Children with chronic conditions or highly specialized healthcare needs often receive fragmented healthcare services [56,57]. In the pathway, we identified gaps in follow-up monitoring, care continuity, and coordination between caregivers, multidisciplinary teams, and post-hospital residential and educational settings (***Table 1***, Gap: 16–29). These care gaps lead to less than optimal care and adverse health outcomes stemming from missed clinic appointments. PPWE with more missed clinic appointments have increased ED utilization and unplanned hospitalizations, and this correlation was ascribed to the fact that the appointments are opportunities for medication changes and serve as reminders for medication adherence at home. Patients with one ED visit or unplanned hospitalization in one year had a mean of 0.37 no-show epilepsy appointments; meanwhile, patients with more than one ED visit or unplanned hospitalization in one year had a mean of 1.78 no-show epilepsy appointments. PPWE were less likely than other children with highly specialized healthcare needs to have coordinated care needs met, specifically regarding receiving comprehensive care from a medical home and having accessible community-based services. Improved care continuity and coordination may help bridge these gaps.

Reasons for failure to attend follow-up visits in outpatient clinics are variable but may be related to socio-economic context [58] and access to specialty care. The importance of improving insurance coverage to guarantee access to specialist care has been addressed in prior studies [59]; nevertheless, implementation of an integrated coordination system could contribute to bridging these gaps. This strategy may reinforce patient and caregiver accountability and cultivate a robust patient-provider relationship required for care continuity. It could expand seizure diary and detection device utilization and adherence, and deliver quality data to assist provider decision-making.

Care coordinators and nurse navigators can also provide similar support and facilitate out-of-hospital care continuity by enabling contact opportunities between families and clinical staff [60,61]. Through a nurse navigator relationship, caregivers can comfortably ask questions and relay concerns [60] that may require follow-up, thus closing the care loop. Not only do patients request this support, but also, when surveyed, coordinators found the greatest value in their roles to be their ability to communicate a SAP and provide additional educational handouts regarding seizure disorders. Although adherence to diary entry, inaccurate timing records, awareness of seizure, and the recording of “false positive” events may limit the correct application of diaries, these challenges could be addressed by strengthening caregiver education and confidence (***Table 2: R & S***).

Although epilepsy treatment and practice guidelines have been established, a lack of training amongst neurologist and epilepsy healthcare providers remain a barrier to standardized care. To characterize care delivery practice patterns and knowledge gaps, a survey was employed to neurologists [62] and a separate survey to PPWE to measure [63] provider adherence to eight American Academy of Neurology Epilepsy Quality Measures [64]. Both surveys determined that gaps exist between the recommended care with adherence to guidelines and the care received by patients, and that further education and training amongst all providers is critical to improving the quality and standardization of care [62,63].

Educational institutions are outside-of-hospital settings for gaps in seizure care. To minimize these gaps, educational institutions require clear information and communication regarding steps to take for seizure management [65]. While RM-specific training bolstered school staff self-confidence and reduced errors for RM administration [24], a SAP with clear guidelines stating who to call in a seizure event, who is responsible for RM administration, and how to identify adverse effects of medications should be outlined, possibly aided by a coordinator [65]. Thus, coordinators can help bridge gaps between healthcare and all out-of-hospital settings (***Table 1***, Gap: 25–29).

An epilepsy-integrated care coordination system that spans all levels (national, federal, state, regional/community, practice, and family) and has designated responsibilities for stakeholders is essential to achieving optimal outcomes. It is important to note that integrated care coordinates between providers across agencies and institutions [66]. The Care Coordination Fund defines a high-performing pediatric coordination framework as one that is “patient-and-family centered, proactive, planned and comprehensive, promotes self-care skills and independence and emphasizes cross-organizational relationships.” As the patient-family is the center of care, their input is critical to the design and implementation of infrastructure and policies. Integrated care strategies must enhance caregiving capabilities of families and address “medical, social, developmental, educational and financial needs.” [67] A three-pronged integrated approach focuses on improving individual care, health at a population level, and reducing healthcare delivery cost [68].

### Prevention of ED Utilization and Hospitalization

Despite a growing awareness for epilepsy preventive care and prompt evidence-based seizure treatment algorithms, there is room for improved integrated seizure care training and counseling for caregivers within healthcare processes. A specialized epilepsy urgent care clinic integrated within the hospital workflow improved caregiver access to education and counseling, and reduced ED visits and unplanned hospitalizations [27,28]. In conjunction with QI initiatives, the urgent clinic contributed to a reduction of ED visits by 28% and unplanned hospitalizations by 43%. Children were also significantly less likely to visit the ED three months following the clinic [27,28]. With a 93% attendance rate for scheduled patients, the clinic offered patients and caregivers extensive time with clinicians. Eliminating barriers through an urgent care clinic and increased caregiver access to comprehensive treatment and psychosocial and educational counseling may lead to optimized care delivery, improved outcomes, and reduced hospital visits.

High ED utilization for PPWE is predictive of continued ED use. A prudent strategy to combat increased hospitalizations would be to identify those with a high risk for increased hospital utilization for seizures and create customized care plans and focused training to prevent adverse events. To determine care steps effectively, researchers implemented a QI dashboard to present patients to the clinic staff that missed outpatient clinic appointments and had increased ED utilization and hospitalizations [28].

### Implementation Science

Clinical effectiveness and implementation research studies are imperative to design and test interventions in the seizure care pathway. The Quality Enhancement Research Initiative (QUERI) developed a core six-step implementation process model that outlines steps for identifying care quality gaps and enhancing the adoption of evidence-based clinical practices and improvements for patient outcomes. QUERI offers researchers proven methodology and processes to overcome implementation challenges [69].

As detailed in QUERI’s Step 3, the process map diagnoses quality and performance gaps, and identifies barriers and facilitators to improvements. The process map also addresses Step 4 which discusses the implementation of improvement programs through identification, development, and implementation of improvement strategies, programs, and tools. Step 5 and 6 evaluate these implementation trials [69].

Through the Seizure Disorder Episodes of Care project, researchers learned lessons fundamental to implementation science, with the first lesson underscoring the importance of patience and an acceptance of staff learning curves. Widespread process improvement required long-term commitment from upper-level management and a supportive and balanced multidisciplinary care team. Researchers noted that effective implementation required that practice guidelines and physician aid tools be clearly written instructions, designed from inception with direct care team input and be fully integrated into care-team daily workflow with minimal burden on workload [70]. Acknowledging that the National Institute of Health has developed funding aims supporting implementation research [71], intervention and knowledge transfer studies are critical for creating effective and meaningful change in the care pathway.

### Limitations

The seizure care pathway may not reflect current practice and challenges faced by hospitals with different processes. Nonetheless, we consider that this comprehensive process map is a necessary first step to identify challenges in our practice that may guide QI projects and ultimately serve as a framework to other centers that aim to improve seizure management. The proposed strategies to address the identified gaps may be difficult to apply due to costs or diverse infrastructures. QI projects are needed to understand the feasibility of these strategies and their impact on seizure management and patient outcomes. We did not assess the combined effects of these strategies on patient care.

## Conclusion

The acute seizure care pathway illustrates current practice, identifies twenty-nine care gaps, and highlights strategies that may improve the quality and efficiency of care delivery for PPWE. The pathway achieves the first step in understanding the cause of care gaps by identifying challenges, especially where current interventions and guidelines have not been integrated into clinical practice. Identification of these gaps provides the foundation for implementing interventions and strategies in seizure management. QI and implementational studies are needed to assess the outlined interventions.

## Author Informations

Tobias Loddenkemper serves on the Council of the American Clinical Neurophysiology Society, as founder and consortium PI of the pediatric status epilepticus research group (pSERG), as an Associate Editor for Wyllie’s Treatment of Epilepsy 6^th^ edition and 7^th^ editions, and as a member of the NORSE Institute, and CCEMRC. He served as Associate Editor of Seizure (Elsevier), and served on the Laboratory Accreditation Board for Long Term (Epilepsy and Intensive Care Unit) Monitoring, and American Board of Clinical Neurophysiology in the past.

He is part of patent applications to detect and predict clinical outcomes, and to detect, manage, diagnose, and treat neurological conditions, epilepsy, and seizures. Dr. Loddenkemper is co-inventor of the TriVox Health technology, and Dr. Loddenkemper, and Boston Children’s Hospital might receive financial benefits from this technology in the form of compensation in the future.

He received research support from the Epilepsy Research Fund, NIH, CIMIT/DoD, PCORI, the Epilepsy Foundation of America, the American Epilepsy Society, the Epilepsy Therapy Project, the Pediatric Epilepsy Research Foundation, the Danny Did Foundation, Cure, the HHV6 Foundation, and received research grants from Lundbeck, Eisai, Upsher-Smith, Mallinckrodt, Sunovion, Sage, Empatica, Acorda, and Pfizer, including past device donations from various companies, including Empatica, SmartWatch, and Neuro-electrics. In the past, he served as a consultant for Eisai, Lundbeck, UCB, Amzell, Sunovion, Upsher Smith and Zogenix.

He performs video electroencephalogram long-term and ICU monitoring, electroencephalograms, and other electrophysiological studies at Boston Children’s Hospital and affiliated hospitals and bills for these procedures and he evaluates pediatric neurology patients and bills for clinical care.

He has received speaker honorariums/Grand Round travel support from national/international societies and national/international academic centers.

Some of Dr. Loddenkemper’s trainees received salary support from international foundations/societies and academic centers while working in his laboratory.

His wife, Dr. Karen Stannard, is a pediatric neurologist and she performs video electroencephalogram long-term and ICU monitoring, electroencephalograms, and other electrophysiological studies and bills for these procedures and she evaluates pediatric neurology patients and bills for clinical care.
